# The mouse viral outgrowth assay: avatars for the detection of HIV-1 reservoirs

**DOI:** 10.1186/s12977-017-0376-z

**Published:** 2017-11-21

**Authors:** Kelly A. Metcalf Pate, Joel N. Blankson

**Affiliations:** 1 Department of Molecular and Comparative Pathobiology, Center for AIDS Research, The Johns Hopkins School of Medicine, Baltimore, MD USA; 2 Department of Medicine, Center for AIDS Research, The Johns Hopkins School of Medicine, Baltimore, MD USA

**Keywords:** Mouse viral outgrowth assay, Reservoir, Latency, HIV-1, SIV, QVOA

## Abstract

Sensitive assays are needed for the detection of residual viral reservoirs in HIV-1-infected subjects on suppressive combination antiretroviral therapy regimens to determine whether eradication strategies are effective. Mouse viral outgrowth assays have recently been developed and have the potential to be more sensitive than traditional in vitro quantitative viral outgrowth assays. In this article we describe these assays and review several studies that have used them to measure the latent reservoir.

## Background

Animal sentinels have been alerting humanity to the presence of infectious disease in their midst since the proverbial “canary in a coal mine”. Such sentinels range from migratory birds in the United States and wild apes in Africa that are routinely screened for the detection of emerging pathogens [1, 2], to laboratory mice in research facilities monitored for the detection of viruses, bacteria and parasites that may confound research results in translational animal models [3]. The medical community has taken advantage of the ability of animals to amplify or respond to human pathogens. Historically, mouse inoculation tests have been key in diagnosing rabies from human tissue samples [4], detecting *Listeria monocytogenes* contamination in food [5], and identifying enterotoxin-producing* Escherichia coli* in human feces [6]. Even into the twenty first century, mouse inoculation assays remained a cornerstone for the diagnosis of *Clostridium botulinum* or *tetani* toxicity until they were recently replaced by quantitative PCR techniques [7].

HIV-1 cannot replicate in any known animal host other than great apes [8, 9]. However, immunodeficient mice that have been xenografted with human immune cells can be productively infected with HIV-1 [10]. Researchers have worked with these mice to learn about key elements of the pathogenesis of HIV-1 infection, including the host immune response and viral evolution, and to evaluate novel antiretroviral drugs, vaccines and cure strategies. The most common humanized mouse models include the peripheral-blood leukocyte (PBL)-engrafted NOD.Cg-Prkdc^scid^IL2rg^tm1Wjl^/SzJ (NSG) mouse and the bone marrow–liver–thymus (BLT) mouse, though many variations of and beyond these exist [11]. All xenografted mouse models (with one notable exception) [12] eventually develop graft-versus host disease. This is the product of immune activation of the human leukocytes in response to the host mouse’s antigen, and results in a cell-mediated immune response characterized by an increase in CD4+ T cell activation, infiltration of the skin and other organs with CD4+ T cells, and sustained production of Th1 cytokines [13].

## Main text

**Fig. 1:**
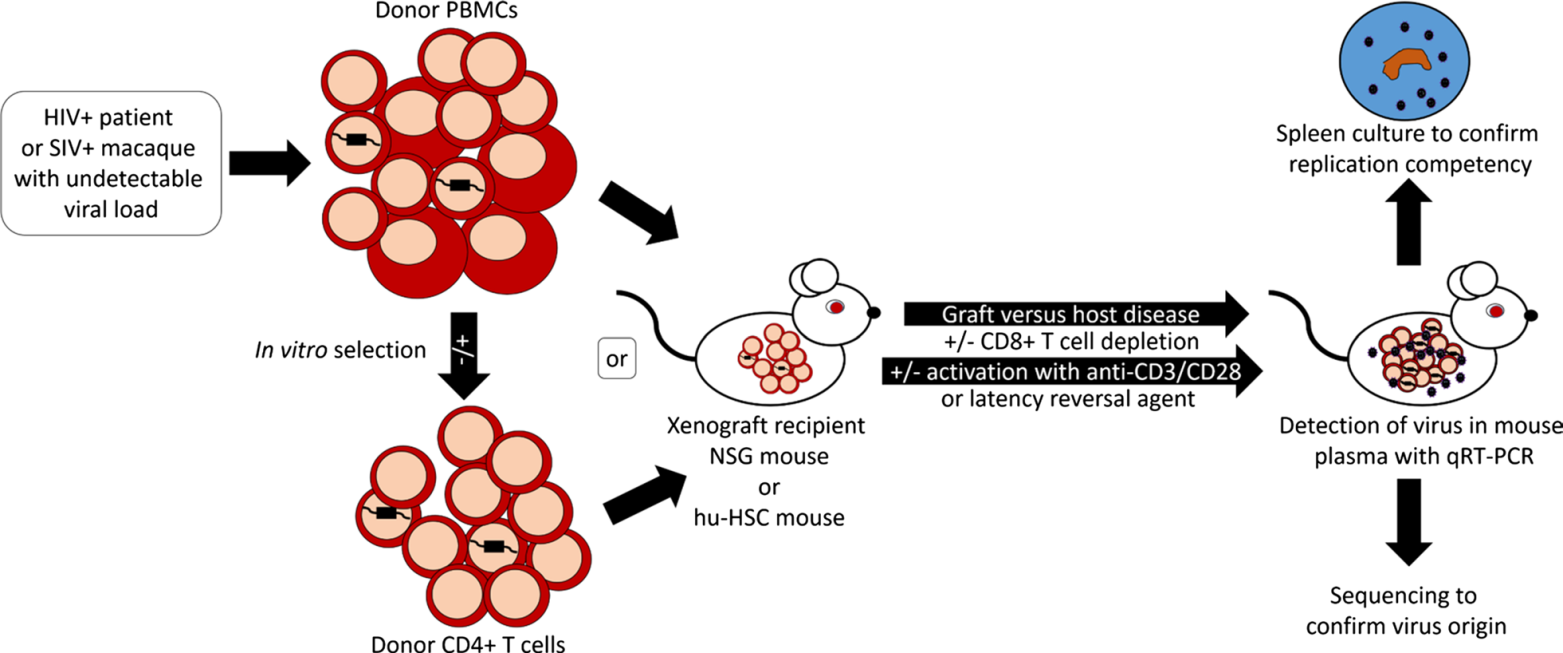
The MVOA amplifies replication competent HIV-1 or SIV following xenograft of samples from subjects or macaques with undetectable viral load. NSG or hu-HSC mice may act as recipient for donor PBMCs or purified CD4+ T cells. Sustained cytokine stimulation secondary to graft versus host disease in the xenografted mouse may be supplemented with exogenous activating anti-CD3 or anti-CD28 antibody treatment or a latency reversal agent, and CD8+ T cells may be depleted in the mouse to decrease targeted killing of infected cells within the xenograft. HIV-1 or SIV may be detected in the mouse plasma by qRT-PCR or other methods. The recipient mouse spleen may be cultured to confirm replication competency, and the virus may be sequenced to confirm origin

The original murine viral outgrowth assay (MVOA) is a variation of the PBL-NSG humanized mouse model, and benefits from this sustained immune response to stimulate the production of latent HIV-1 from HIV-1-infected subjects’ tissue. Peripheral blood mononuclear cells (PBMCs) or purified CD4+ T cells from infected subjects with undetectable plasma viral loads become activated and release replication competent virus after xenograft into immunocompromised NSG mice via intraperitoneal injection [14]. Activation is evidenced by increased CD25, CD69 and HLA-DR expression on xenografted CD4+ T cells. In the murine host, qRT-PCR can be used to quantify plasma HIV-1 RNA released from the xenografted cells [14]. Positive results can be verified by culturing spleen cells from the xenografted mouse to confirm the production of replication competent virus that is homologous to virus amplified from the subject [15]. While effective engraftement is routinely seen in our hands when 20 million or more PBMCs or purified CD4+ T cells per mouse are used, we have engrafted as many as 50 million cells per mouse. However, progression to terminal graft versus host disease accelerates significantly when over 50 million cells are xenografted [14]. Strategies for improving the sensitivity of the assay include depleting xenografted CD8+ T cells and stimulating CD4+ T cells in vivo with exogenous activating anti-CD3 and/or anti-CD28 antibody or latency-reversing agents (Fig. 1) [14].

In the original report, these techniques allowed for the detection of virus in mice engrafted with cells from 5 of 5 subjects with undetectable plasma viral loads on anti-retroviral therapy (ART), and 6 of 6 elite suppressors with undetectable plasma viral loads, including one who was undetectable by quantitative viral outgrowth assay (QVOA). Peak viral loads in the MVOA ranged from 2.3 × 10^3^ to 1.7 × 10^7^ copies/mL within 13–26 days after xenograft for subjects on ART, to 1.1 × 10^3^ to 3.8 × 10^5^ copies/mL within 4–49 days after xenograft for elite suppressors [14].

More recently, the MVOA was used to study samples from two subjects who were started on ART in the very early stages of primary infection. Subject A was infected approximately 10 days before starting pre-exposure prophylaxis (PrEP) with tenofovir disoproxil and emtricitabine. A full ART regimen was initiated after 7 days of PrEP when his viral load was a mere 220 copies/mL. Low level cell-associated HIV-1 RNA (3.2 copies/million CD4+ T cells) was detected on day 32 after infection, but over the next 2 years no HIV-1 DNA, RNA or replication-competent virus was detected from PBMCs or cells isolated from the ileum, rectum, lymph nodes, bone marrow and cerebrospinal fluid using PCR, RNA inducibility assays, and the traditional quantitative viral outgrowth assay (QVOA). Peripheral CD4+ T cells were assayed in the MVOA and 1 of 10 mice that were each xenografted with 53 million cells developed a viral load of 201 copies/mL at 5.5 weeks [16]. The participant eventually stopped ART and remained aviremic for 7.4 months before rebounding with a viral load that eventually rose to 59,805 copies/mL. Subject B was infected approximately 12 days before initiating PrEP and was started on a full ART regimen on day 12 of PrEP when he had a peak viral load of 3343 copies/mL. No replication competent virus was produced when 20 million CD4+ T cells were cultured in a QVOA, but when 50 million cells were xenografted into each of 8 mice, viral loads of 1000, 5000, and 11,000 copies/mL respectively were detected in 3 mice. ART has not been discontinued in this study participant [16]. While we were not able to sequence the plasma virus from any of the viremic mice to prove that the viral isolates were subject-specific, the two cases illustrate the sensitivity of the MVOA, its ability to assay very large numbers of subject cells relatively easily and its potential utility as an avatar for the individual subject who is considering discontinuation of ART.

Salgado and colleagues similarly used the MVOA to xenograft cells from 6 subjects with hematalogic malignancies who had allogeneic stem cell transplantation as part of their treatment [17]. Prior studies have shown that these subjects can have very low numbers of latently infected CD4+ T cells as determined by the traditional QVOA [18]. One of the 6 participants had a low level but positive QVOA when large amounts of CD4 T cells were tested, while samples from the other individuals were negative by all the methods used to measure the size of the HIV-1 reservoir. None of the 6 participants had a positive MVOA (5 mice tested per donor, infusion of 10–50 million cells). At the time that this data were presented, these 6 subjects remained on ART so further studies will be needed to determine whether this lack of detection of virus was due to a very low frequency of latently infected cells or to the assay not being sensitive enough to detect these rare cells.

A variation of the MVOA was recently used to determine whether CD4+ T cells from a negative viral outgrowth assay could produce virus in vivo [19]. Li and colleagues xenografted NSG mice with cells from either a negative or positive QVOA well from the same subject. Unfractionated cells from the subject were xenografted as a positive control. Interestingly, while plasma virus was detected 4 weeks after engraftment of a mouse with the positive QVOA cells, the mouse xenografted with cells from the negative QVOA did not become viremic until 10 weeks after engraftment. The results from this proof of concept study are consistent with data that suggests that latency reactivation is a stochastic process and repeated stimulation can lead to latency reversal in cells that did not initially produce virus in the QVOA [20, 21]. Additional work is needed to determine if the time to viremia in the MVOA may correlate with the size of the latent reservoir.

Charlins and colleagues developed a related humanized mouse viral outgrowth assay (hmVOA) using BLT humanized mice [22]. These mice have human lymphocytes present at baseline due to the presence of human fetal thymic tissue that allows for T cell maturation [23]. In the hmVOA, CD4+ T cells from subjects on suppressive ART regimens were stimulated overnight and then injected into BLT mice at limiting dilutions (0.1–20 million CD4+ T cells per mouse). A simultaneous traditional QVOA was performed in order to compare the sensitivity of the 2 assays. Plasma virus was successfully obtained from mice inoculated with cells from 6 subjects where the traditional QVOA was also positive. The investigators further tested the sensitivity of the hmVOA with 5 subjects whose CD4+ T cells did not produce virus in the traditional QVOA. Inoculation of CD4+ T cells from 4 out of 5 of these subjects into BLT humanized mice resulted in the detection of virus.

The MVOA is not limited to human cells; it also has the potential to improve the detection of simian immunodeficiency virus (SIV) in macaque models when evaluating promising vaccine or cure regimens. Additional techniques are available for the detection of SIV in macaques on preclinical trials to supplement QVOA and PCR based assays: Adoptive transfer, the practice of transplanting cells (typically harvested from lymph nodes) from an infected macaque donor into a naïve uninfected macaque recipient, is considered the penultimate technique for the detection of latent reservoirs in macaque models, and release from ART, the practice of ceasing therapy to determine whether virus will rebound, is the gold standard [24]. However, because of the high value and the limited availability of macaques, highly sensitive alternative methods of detecting residual virus are needed. The MVOA can detect replication competent SIV after xenograft of PBMCs or purified CD4+ T cells from a pigtailed macaque (*Macaca nemestrina*) model of HIV-1 latency [14, 25]. In the original report, MVOA successfully amplified SIV from PBMCs and CD4+ T cells from a macaque that had a 78 day duration of undetectable plasma viral loads and had been on ART for 193 days, with a peak viral load of 1.3 × 10^4^ copies/mL detected in the mouse within 7 days of xenograft. That macaque was the only animal with undetectable viral load evaluated in the study, though 4 additional viremic animals were also successfully screened by MVOA [14]. Additional work is needed to determine if the MVOA could serve as a valuable adjunct to existing assays for the detection of latent SIV in macaque models, or replace the costly practice of adoptive transfer into naïve macaques. It would be further advantageous to evaluate if the MVOA can be used to detect latent SIV in lymph nodes and other tissues, as such a modified assay could be used to define novel sanctuary sites and latent viral reservoirs in addition to more rigorously testing putative preventative and cure strategies.

## Conclusions

Many different assays have been developed to measure the latent reservoir. Each of these assays has its strengths and limitations. While the QVOA is the gold standard for the detection of replication-competent virus, it is not particularly sensitive. The mouse viral outgrowth assays are capable of easily assaying a very large number of cells for replication-competent virus compared to the traditional QVOA that has a requirement for a tenfold excess of irradiated feeders [26]. Another advantage of these assays is that the viral load can be quantified, and the degree of change in this parameter over time may reflect the degree of fitness of the replicating virus. Thus the mice in these assays may be used as clinical avatars for subjects before decisions are made about treatment interruptions.

The MVOA in particular recapitulates what happens when a subject stops treatment with the added advantage of rapid activation of the majority of the subject’s cells by graft versus host disease. In contrast, the presence of mature human lymphocytes at baseline in BLT mice in the hmVOA means that there is likely to be both graft versus host and graft versus graft disease after the inoculation of the subjects’ CD4+ T cells. The donor cells may not survive for long periods of time, but it is likely that any virus produced by the activation process will be expanded in the host BLT mouse’s human CD4+ T cells. While the hmVOA has the advantages of being able to assay as few as 0.1 million subject cells and having longer engraftment times due to a much lower degree of GVHD, the higher cost of these mice as well as the requirement for human fetal tissue makes the original MVOA easier to work with. The original MVOA can also be used for both HIV-1 and SIV studies whereas the hmVOA is limited to studying HIV-1 because of the engraftment of human tissue. A recent study has also suggested that the selective engraftment of human memory CD4+ T cells significantly delays the onset of GVHD in the MVOA allowing for longer periods of time for viral rebound to occur [27]. A disadvantage of the MVOA is that it is not as quantitative as the QVOA, but the percentage of engrafted mice that become viremic [16] and the time to viral rebound [16, 19] can potentially provide clues about the size of the reservoir. Additional work is needed to further define and increase the sensitivity of the MVOA, including the examination of whether supplementation of the sustained activation provided by the graft versus host disease with pre- (in vitro) or post-xenograft (in vivo) anti-CD3/CD28 activating antibody and/or latency reversing agents, or elimination of CD8+ T cells pre- or post-xenograft, will increase the yield of virus.

In summary, the mouse viral outgrowth assays are sensitive in vivo assays that specifically measure replication-competent virus. They can be used to interrogate very large numbers of cells and thus they may be better able to detect residual virus in subjects with very small reservoirs even if their intrinsic sensitivity is not higher than that of the QVOA. The two subjects who were started on PrEP within days of infection perhaps best illustrate this point. The mouse viral outgrowth assays may be most useful when other assays are negative and a large number of cells need to be tested for replication-competent virus prior to treatment interruption. Although initial results are encouraging, the assays need to be validated with a large number of samples, especially those that are negative by other replication-competent virus measurements.

## Abbreviations

MVOA: mouse viral outgrowth assay, also referred to as murine viral outgrowth assay
hmVOA: humanized mouse viral outgrowth assay
QVOA: quantitative viral outgrowth assay
NSG: NOD.Cg-Prkdc^scid^IL2rg^tm1Wjl^/SzJ
BLT: bone marrow, liver, thymus
cART: combination antiretroviral therapy
PBMC: peripheral blood mononuclear cell
qRT-PCR: quantitative real-time polymerase chain reaction
Th1: type 1 T helper cell
HIV-1: human immunodeficiency virus-1
SIV: simian immunodeficiency virus
